# FACTORS INFLUENCING OLDER KOREAN AMERICAN IMMIGRANTS’ FRAILTY: A QUALITATIVE STUDY

**DOI:** 10.1093/geroni/igad104.3344

**Published:** 2023-12-21

**Authors:** Hae Sagong, Myoung-Gi Chon, Pao-Feng Tsai

**Affiliations:** Auburn University, Auburn, Alabama, United States; Auburn University, Auburn, Alabama, United States; Auburn University, Auburn, Alabama, United States

## Abstract

The older immigrant population, characterized by unique attributes such as diverse cultural backgrounds, health beliefs, health behaviors, language barriers, and low health literacy, requires a specific focus on identifying factors that influence frailty. This study aims to explore the influencing factors of frailty among older Korean American immigrants (OKAIs) residing in suburban areas with limited Korean healthcare infrastructure. Semi-structured interviews were conducted with 9 OKAIs residing in Lee County, Alabama, focusing on four factors associated with frailty: health literacy, physical activity, diet, and mental health. Qualitative content analysis was employed to analyze the data. Validated assessment tools for frailty, health literacy, physical activity, nutrition, depression, and cognitive impairment were also used to gather participant characteristics and complement the qualitative findings. Participants exhibited significant differences in health literacy scores between the Korean and English versions. Content analysis yielded seven categories and twenty-two sub-categories were derived from the eighty identified codes. In addition to typical age-related changes observed among older adults in general, their social activities, health behaviors, and healthcare visits were constrained by different environments/healthcare systems compared to Korea, as well as limited English proficiency and healthcare resources. Participants expressed a need for Korean healthcare providers or interpreters during their healthcare visits and emphasized their preference for Korean food and sought ways to maintain vitality. To prevent and manage frailty among OKAIs, interventions need to consider their unique characteristics, facilitators, and barriers. This approach may also benefit other ethnic minority populations facing similar challenges, thus enhancing healthcare for these communities.

